# The association between post-term births and autism spectrum disorders: an updated systematic review and meta-analysis

**DOI:** 10.1186/s40001-023-01304-2

**Published:** 2023-09-02

**Authors:** Ensiyeh Jenabi, Sajjad Farashi, Amir Mohammad Salehi, Hamideh Parsapoor

**Affiliations:** 1 Autism Spectrum Disorders Research Center, Hamadan University of Medical Sciences, Hamadan, Iran; 2 Student Research Committee, Hamadan University of Medical Sciences School of Medicine, Hamadan, Iran; 3 Clinical Research Development Unit of Fatemieh Hospital, Department of Gynecology, Hamadan University of Medical Sciences, Hamadan, Iran

**Keywords:** Post-term, Autism spectrum disorders, Meta-analysis study

## Abstract

**Background:**

This study aimed to conduct a meta-analysis to determine whether post-term birth has an increased risk of ASD.

**Materials and methods:**

To retrieve eligible studies regarding the effect of post-term and ASD in children, major databases including PubMed, Scopus, and Web of Science were searched. A random effect model was used for meta-analysis. For assessing the quality of included studies, the GRADE checklist was used.

**Results:**

In total, 18 records were included with 1,412,667 sample populations from 12 countries. The pooled estimates of RR and OR showed a significant association between post-term birth and ASD among children, respectively (RR = 1.34, 95% CI 1.10 to 1.58) and (OR = 1.47, 95% CI 1.03 to 1.91). There was no heterogeneity among the studies that reported the risk of ASD among children based on RR (*I*^2^ = 6.6%, *P* = 0.301). There was high heterogeneity in the studies reported risk of ASD based on OR (*I*^2^ = 94.1%, *P* = 0.000).

**Conclusion:**

Post-term births still occur relatively frequently (up to 5–10%) even in developed countries. Our results showed that post-term birth is an increased risk of ASD, although high heterogeneity was found among the studies reported based on adjusted and crude forms, however, after subgroup analysis by gender, this heterogeneity disappeared among males.

## Introduction

Autism spectrum disorder (ASD) is defined by severe social communication deficits and stereotyped, repetitive behaviors. Recognizing that the social problems that characterize ASD can occur in different forms, depending on language skills, general ability level, the severity of symptoms, context, and coexistence disorders, has led to a dramatic increase in the number of children with ASD [1].

The pathophysiology of ASD is not fully understood, ASD was previously thought to be mainly genetic, however, genetic factors alone have been found to account for 20–30% of ASD cases, whereas the remaining 70–80% are due to a complex interaction between environmental risk factors such as prenatal and postnatal environments and genetic predisposition [2].

A post-term birth extends beyond 42 weeks (294 days) from the first day of the last menstrual period, although the routine use of ultrasound to confirm the date of pregnancy can reduce the incidence of post-term birth, however, post-term birth still occur relatively frequently (up to 5–10%) even in developed countries [3] and associated with neonatal morbidity and mortality [4].

A prior systematic review has reported an overall prevalence of 0.6% of ASD in people with post-term birth [5]. Some studies have reported post-term birth as a pregnancy risk factor for ASD [6, 7], while other studies have not shown this relationship [8, 9], also previously, meta-analysis found that post-term birth is not a risk factor for ASD [10]. Therefore, this study aimed to conduct a meta-analysis to determine whether post-term birth has an increased risk of ASD.

## Materials and methods

**Fig. 1:**
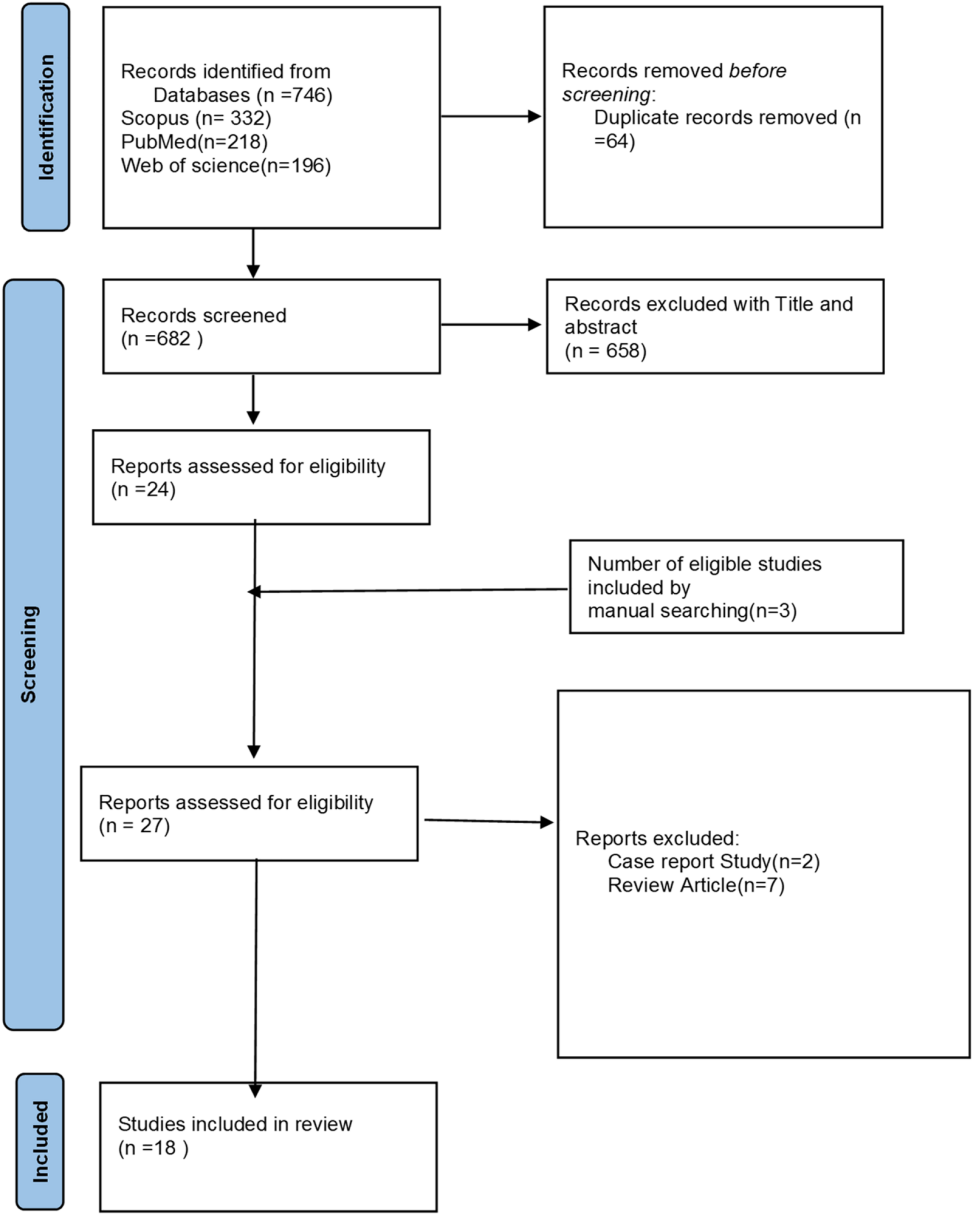
Flowchart of the selection process of the studies in the different phases of the meta-analysis

This meta-analysis was performed considering the Preferred Reporting Items for Systematic Reviews and Meta*-*Analysis (PRISMA) guidelines [11]. The number of retrieved sources, the number of excluded sources, and the reason for exclusion are given in the PRISMA diagram (see Fig. 1).

### Search procedure

To retrieve eligible studies regarding the effect of post-term birth on ASD, major databases including PubMed, Scopus, and Web of Science were searched by the following keywords: post-term OR postterm OR postmature*) and (autism OR autistic OR autism spectrum disorders OR ASD).

### Inclusion and exclusion criteria

Only original research articles were considered and other types of articles including letters to the editor, case reports, and other systematic reviews were excluded. However, the reference list of such papers was investigated carefully for finding missed sources during database searching. Studies focused on the causal relationship between ASD and post-term birth including cases was included. There was no restriction on the language or the publication date of retrieved sources. Furthermore, only peer-reviewed articles (in published or in-press status) were considered.

### Study selection and data extraction

Two independent authors (A.S and E.J) performed the search procedures. Any conflict regarding the retrieved sources was resolved by discussion between authors. A PECOS (Participant, Exposure, Comparison, Outcome and Study design) model was used for finding eligible studies. The Participants were all newborns, the Exposure was post-term birth, the Comparison was term birth, the Outcome was ASD and the Study design was observational study. After the title, abstract and full-text screening and adding the retrieved sources found by manual searching or found by checking the reference list of eligible studies, an electronic form was used for extracting information embedded in each source. The information included the first author's name, publication date, the country that the study was performed, study design, sample size, the measure that was used for effect size estimation, the age range of participants, diagnostic criteria that were used for autism, and quality of each study.

### Quality assessment

The evaluation of the studies that were included in the analysis was conducted using a tool called the Grades of Recommendation, Assessment, Development, and Evaluation (GRADE) [12]. The assessment considered several important factors including limitations, consistency, directness, imprecision, reporting bias, strength, gradient, and confounding.

### Sensitive analysis

In situations where there was high between-study heterogeneity, we employed a sequential algorithm [13] to determine the source of the heterogeneity. This involved removing one study from the calculations at a time and identifying which study had the greatest impact on decreasing *I*^2^. This process was repeated for a new set of n-1 studies, and continued until *I*^2^ dropped below the intended threshold of 50%. If there were multiple studies that could potentially result in *I*^2^ dropping below the threshold, we reported the minimum *I*^2^.

### Statistical analysis

A random effect model was used for meta-analysis. The effect size was calculated using the odds ratio (OR) and relative risk (RR) with a 95% confidence interval (where the risk increases the occurrence of a desired outcome, the OR and RR will be greater than 1). All analyses were performed using STATA, version 13 (StataCorp, College Station, TX, USA). Furthermore, for all statistical analyses, the significance level was adjusted to 0.05. To investigate the source of heterogeneity, subgroup analyses for the type of study design, gender, and quality of studies were also performed.

The between-study heterogeneity was calculated based on *I*^2^ statistic [14] and Cochrane Q-test. The value obtained for *I*^2^ determines the heterogeneity between studies in a way that for *I*^2^ > 50% a significant between-study heterogeneity exists; otherwise, when *I*^2^ merges to zero, between-study heterogeneity is non-significant. The Cochrane Q-test determines the statistical significance of the heterogeneity. For assessing publication bias, the funnel plot was used as a graphical tool. The symmetrical distribution of studies in a funnel plot implies the absence of publication bias. Furthermore, Egger's [15] and Begg's [16] tests were used as quantitative measures for assessing publication bias.

## Results

### Description of studies

In total, 746 records were identified by initial search (Fig. 1). We removed duplicates, and then 682 records were retained for more evaluation. Subsequently, 655 ineligible records were excluded by reviewing the titles and abstracts. The remaining 27 full paper records were evaluated for eligibility, of which nine records were excluded. Eventually, a total of 18 records (six cohorts [8, 17–21] and 11 case–controls [6, 7, 9, 22–29], and one cross-sectional study [30]) were included with 1,412,667 sample populations from 12 countries (Table 1).

**Table 1:** Summary results of the included studies

1st author, year	Country	Design	Sample size	Estimate	Adjustment	Age range (year)/mean	Autism criteria
Brumbaugh, 2020	USA	Cohort	7876	Hazard ratio	Adjusted/crude	3–21	DSM-IV
Leavy, 2012	Canada	Cohort	218110	Odds ratio	Crude	No data	ICD-9
Martini, 2022	Netherland	Case–control	1199	Odds ratio	Crude	14.01	DSM-IV/ DSM-5
Xie, 2017	Sweden	Cohort	480728	Odds ratio	Crude	No data	ICD-9/ICD10
Atladottir, 2016	Denmark	Cohort	519692	Odds ratio	Crude	1–33	ICD-8/ICD-10
Persson, 2020	Sweden/Finland/ Norway	Cohort	50816	Relative risk	Adjusted	2 years until diagnosis	ICD-10
Sugie, 2005	Japan	Case–control	1805	Odds ratio	Crude	3 years or older	DSM-IV
Tawfeeq, 2016	Iraq	Case–control	200	Odds ratio	Crude	3–15	No data
Zhang, 2010	China	Case–control	190	Odds ratio	Crude	3–21	ICD-10/CARS
Fernandes, 2016	India	Cross-sectional	184	Odds ratio	Crude	No data	No data
Al-Ali, 2021	Iraq	Case–control	276	Odds ratio	Crude	1–20	No data
Hisle-Gorman, 2018	USA	Case–control	35040	Odds ratio	Crude	2–8	ICD-9
Laxer, 1988	USA	Case–control	38182	Odds ratio	Crude	No data	DSM-III
Rolschau, 2020	Denmark	Cohort	57888	Odds ratio	Crude	No data	ICD-10
Cryan, 1996	Ireland	Case–control	98	Odds ratio	Crude	4–35	DSM-III
Lord, 1989	USA	Case–control	100	Odds ratio	Crude	3–8	CARS
Gillberg, 1983	USA	Case–control	50	Odds ratio	Crude	7 years or older	No data

### Main analysis

**Fig. 2:**
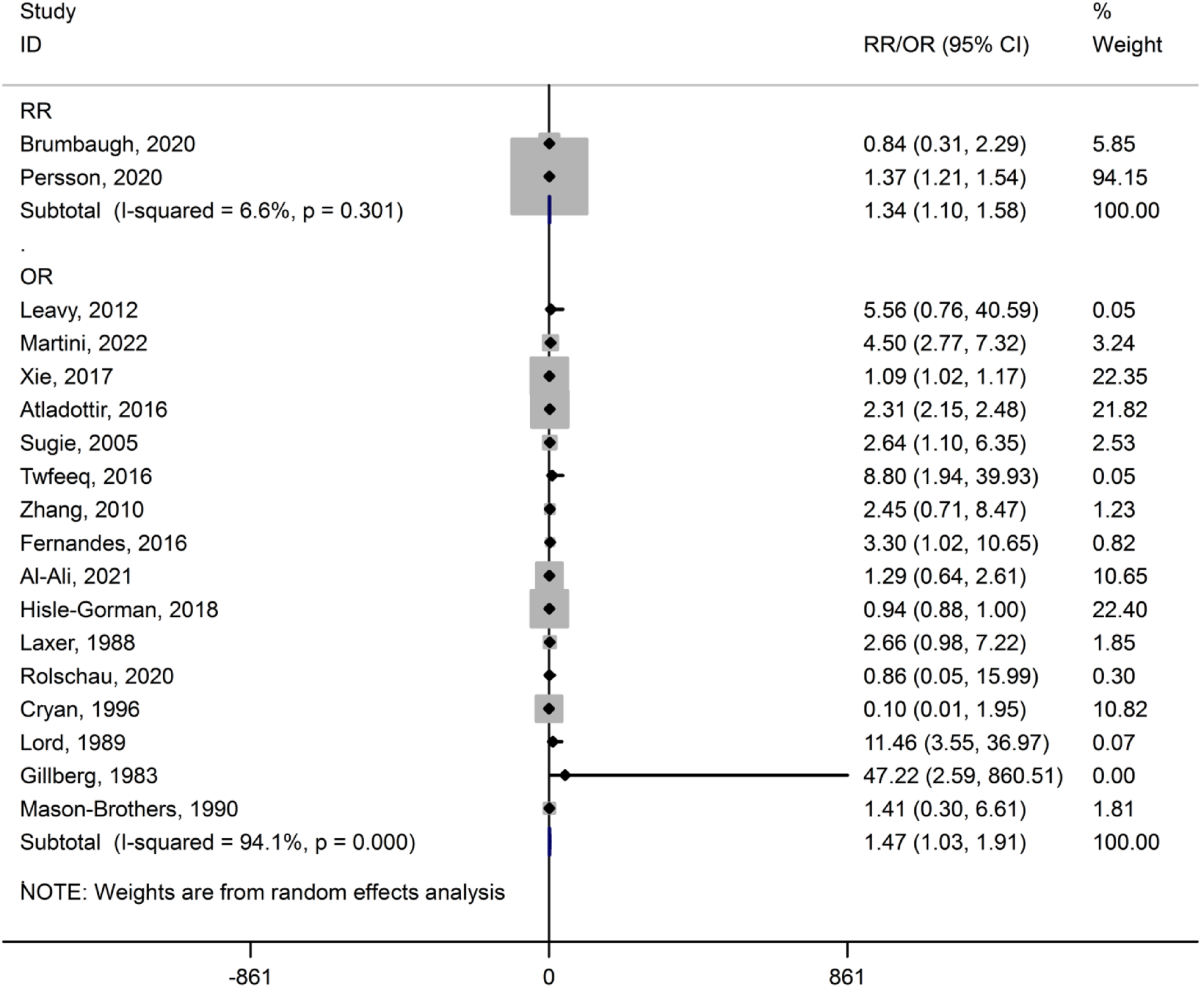
Forest plot of the association between post-term birth and ASD

The association between post-term birth and ASD is presented in Fig. 2. The pooled estimates of RR and OR showed a significant association between post-term birth and ASD, respectively (RR = 1.34, 95% CI 1.10 to 1.58) and (OR = 1.47, 95% CI 1.03 to 1.91). There was no heterogeneity among the studies that reported the risk of ASD based on RR (*I*^2^ = 6.6%, *P* = 0.301). There was high heterogeneity in the studies reported risk of ASD based on OR (*I*^2^ = 94.1%, *P* = 0.000).

**Fig. 3:**
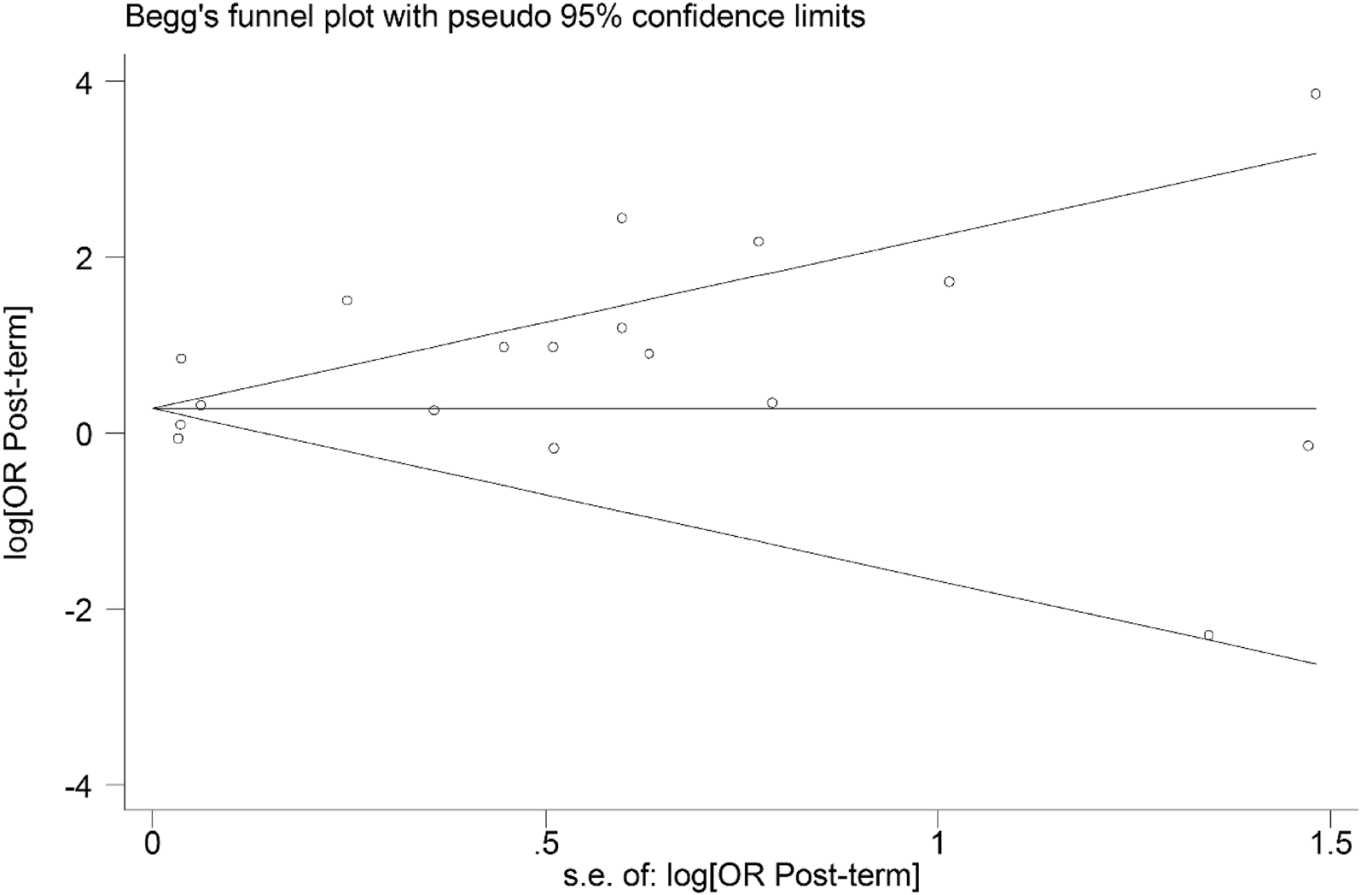
Funnel plot of the association between post-term birth and ASD

The funnel plot is presented in Fig. 3. There was no evidence of publication bias among studies. The *P* values were 0.910 and 0.341 based on Begg’s and Eggerʼs regression, respectively.

### Quality of the studies

The results of this evaluation can be found in Table 2, where it shows that the quality of 5 studies was considered moderate, while 13 studies were assessed as having low quality (Table 2).

**Table 2:** Quality assessment included meta-analysis the association between post-term birth and ASD

Studies	Design^a^	Limitation^b^	Consistency^c^	Directness^d^	Inmprecision^e^	Reporting bias^f^	Strength^g^	Gradient^h^	Counfounding^i^	Quality
Brumbaugh, 2020	Cohort	0	− 1	0	0	0	0	0	+ 1	Low
Leavy, 2012	Cohort	0	− 1	0	0	0	+ 2	+ 1	0	Low
Martini, 2022	Case–control	0	0	0	0	0	+ 1	+ 1	0	Moderate
Xie, 2017	Cohort	0	0	0	0	0	0	+ 1	0	Moderate
Atladottir, 2016	Cohort	0	0	0	0	0	+ 1	+ 1	0	Moderate
Persson, 2020	Cohort	0	0	0	0	0	0	+ 1	+ 1	Moderate
Sugie, 2005	Case–control	0	0	0	0	0	+ 1	+ 1	0	Moderate
Tawfeeq, 2016	Case–control	− 1	0	0	0	0	+ 2	+ 1	0	Low
Zhang, 2010	Case–control	0	− 1	0	− 1	0	+ 1	+ 1	0	Low
Fernandes, 2016	Cross-sectional	− 1	0		− 1	0	+ 1	+ 1	0	Low
Al-Ali, 2021	Case–control	0	− 1	0	0	0	0	+ 1	0	Low
Hisle-Gorman, 2018	Case–control	0	− 1	0	0	0	0	0	0	Low
Laxer, 1988	Case–control	− 1	− 1	0	0	0	+ 1	+ 1	0	Low
Rolschau, 2020	Cohort	0	− 1	0	0	0	0	0	0	Low
Cryan, 1996	Case–control	− 1	− 1	0	− 1	0	+ 2	0	0	Low
Lord, 1989	Case–control	− 1	0	0	− 1	0	+ 2	+ 1	0	Low
Gillberg, 1983	Case–control	− 1	0	0	− 1	0	+ 2	+ 1	0	Low
Mason-Brothers, 1990	Case–control	− 1	− 1	0	0	0	0	+ 1	0	Low

Quality of evidence and definitions: high quality: further research is very unlikely to change our confidence in the estimate of effect; moderate quality: further research is likely to have an important impact on our confidence in the estimate of effect and may change the estimate; low quality: further research is very likely to have an important impact on our confidence in the estimate of effect and is likely to change the estimate; Very low quality: any estimate of effect is very uncertain

^a^Refers to the basic study design, which we have broadly categorized as randomized trials (high), observational (cohort/case–control) studies (low), and other evidence (very low)

^b^Refers to the detailed study methods and execution [serious (− 1) or very serious (− 2) limitation]

^c^Refers to the similarity in the estimates of effect across studies [important inconsistency (− 1)]

^d^Refers to the extent to which the ‘people’, ‘interventions’, and ‘outcome measures’ are similar to those of interest [some (− 1) or major (− 2) uncertainty about directness]

^e^Refers to imprecise or sparse data (− 1)

^f^Refers to the high risk of reporting bias (− 1)

^g^Refers to the strong (relative risk > 2 or < 0.5; + 1) or very strong (relative risk > 5 or < 0.2; + 2) evidence of association with no plausible confounders

^h^Refers to evidence of a dose–response gradient (+ 1)

^I^Refers to all plausible confounders that would have reduced the effect (+ 1)

### Sensitive analysis

To ensure between-study homogeneity, we conducted a sensitivity analysis using the sequential algorithm. By omitting the two studies [20, 23] from the meta-analysis examining the association, we were able to achieve the minimum desired *I*^2^ threshold of 50% (OR = 1.39, 95% CI 0.76, 2.02, *I*^2^ = 30.7%).

### Subgroup analysis

The subgroup analysis was performed based on the design of the studies, the quality of the studies, the child's gender, publication year, continent, and autism diagnosis criteria. OR in case–control and cohort studies were reported 0.94 (0.88, 1.0) and 1.30 (1.23, 1.37), respectively. A significant association was found between post-term birth and ASD among cohort studies (Table 3). In addition, there was a significant association between post-term birth and ASD in the studies with high quality (OR = 1.63, 95% CI 1.11, 2.15). However, this association had high heterogeneity (*I*^2^ = 97.5%). There was no significant association between post-term birth and ASD in the studies with low quality (OR = 0.94, 95% CI 0.88, 1.00). The subgroup analysis based on the child's gender showed that a significant association was found between post-term birth and ASD among male children (OR = 1.37, 95% CI 1.19, 1.56). This association was homogenous, while there was no significant association between post-term birth and ASD among female children (OR = 1.13, 95% CI 0. 84, 1.43). There was a significant association between post-term birth and ASD in the studies that publication year was after 2000 (OR = 1.61, 95% CI 1.13, 2.09). There was not a significant association between post-term birth and ASD in the studies based on continent and autism diagnosis criteria (Table 3). There was significant difference in ASD risk between study design in cohort and case–control studies, quality of the studies between low and moderate quality, child’s gender between male and female and autism diagnoses criteria between ICD and DSM (*P*_interaction_ = 0.001, 0.001, 0.024 and 0.026), but there was no significant difference in publication year between before and after 2000 and continent between Europe/USA and Asian (all *P*_interaction_ = 0.383 and 0.778).

**Table 3:** Results of subgroup analysis of post-term birth and autism spectrum disorders (ASD) based on OR

Subgroups	No. of studies	OR (95% CI)	*P* for *Z* test	*I*^2^ (%)	*p* for interaction
Study design					
Cohort	4	1.30(1.23, 1.37)	0.005	98.3	0.001
Case–control	11	0.94 (0.88, 1.0)	< 0.001	45.8	
Quality of the studies					
Moderate	4	2.19 (1.14, 3.24)	< 0.001	98.4.0	0.001
Low	12	0.94 (0.88, 1.0)	< 0.001	0.0	
Child’s gender					
Male	3	1.37(1.19, 1.56)	< 0.001	0.0	0.024
Female	3	1.13(0. 84, 1.43)	< 0.001	0.0	
Publication year					
Before 2000	5	0.73 (− 0.55, 2.0)	0.262	95.9	0.383
After 2000	11	1.61 (1.13, 2.09)	< 0.001	11	
Continent					
Europe/American	11	1.42 (0.94, 1.90)	< 0.001	96	0.778
Asian	5	1.59 (0.7, 2.47)	< 0.001	0.0	
Autism diagnoses criteria					
ICD-8/ICD-9/ ICD-10	6	1.64 (0.93, 1.99)	< 0.001	97.0	0.026
DSM-III, DSM-IV/ DSM-5	5	2.14 (0.2, 4.08)	0.031	73.3	

## Discussion

The existence of cognitive and learning problems in a person with ASD, its negative impact on patient's social interactions with the world around them, and the increasing cost to the health system have caused researchers to pay much attention to this disorder and work on the factors affecting the disease [31].

There is evidence that preterm birth [32], small for gestational age [33], prenatal and antenatal depression [34], and labor induction [35] are risk factors for ASD, also, some studies have shown that low maternal residential greenness exposure increases the risk of mental disorders such as ASD. The neonatal risk factors such as not breast feeding [36], low weight, jaundice, congenital heart disease, low birth weight, very low birth weight, and small size for gestational age are increase risk of ASD [33, 37–39]. The results of this study showed that post-term birth should be added to the above as a risk factor for ASD.

Although there is insufficient evidence to implicate any one perinatal or neonatal factor in autism etiology, the studies using optimality scales provide some evidence to suggest that exposure to multiple neonatal complications may increase autism risk. It also is important to note that the observed association between perinatal and neonatal complications and the risk of ASD may reflect the consequences of previous prenatal complications [40].

Previously, only a meta-analysis to date has been conducted about the association between post-term birth and the risk of ASD. They showed that post-term birth was not a risk factor for ASD risk (OR = 1.14; 95% CI 0.58 to 2.24) [10]. However, databases were searched until March 2007 and authors had not included the database of the Web of Science. Therefore, the findings of this meta-analysis are required to be updated. In the present meta-analysis, post-term birth is a risk factor for ASD (RR = 1.34, 95% CI 1.10 to 1.58) and (OR = 1.47, 95% CI 1.03 to 1.91).

Post-term birth contributes to severe morbidities for the mother and child, including macrosomia, shoulder dystocia, birth injury, fourth degree perineal laceration, fetal compromise, antenatal and postpartum hemorrhage, fetal dysmaturity, labor  > 24 h and newborn respiratory distress syndrome [41].

Post-term birth is considered a complex biological process, which is related to factors such as the duration of pregnancy, the delivery, the fetus status of the intrauterine, and the gender and fetal–placental system. Studies showed its prevalence in Austria, Belgium, Germany, Turkey, and Denmark to be 0.4%, 0.6%, 2.3%, 0.76%, and 8.1%, respectively, this rate varies between 1 and 2.5% in America and Canada and 1.16% in China [42].

In some studies, it has been found that the occurrence of post-term birth is more common in female fetuses [43]. However, some studies have not reported a significant difference in the occurrence of post-term birth between genders [42]. Differences in the diagnosis of post-term birth and potential racial differences can lead to varying results in studies [42]. Additionally, the sex ratio may be influenced by environmental factors such as wars and natural disasters [44, 45]. Also, due to the high prevalence of ASD in males, it is possible to justify the heterogeneity between studies to some extent.

Post-term birth and ASD have not yet been fully elucidated, and there are several reasons and pathways for it. First, a post-term birth typically has a higher risk for perinatal problems such as prolonged labor, cephalopelvic disproportion, and shoulder dystocia, which are associated with perinatal oxygen deficiency followed by neurobehavioral problems [46]. A second explanation is uteroplacental insufficiency: a non-optimal ‘old’ placenta offers fewer nutrients and less oxygen than a term fetus requires. The lack of nutrients and oxygen may predispose to abnormal fetal development and this may lead to abnormal neurobehavioral development [47].

Thirdly, it is possible that a disturbance of the ‘placental clock’, which regulates the duration of pregnancy, is involved. A marker of this clock is the placental secretion of corticotrophin-releasing hormone, which is lower in post-term deliveries than in term deliveries, is a sign of this clock, and its principal regulator of the maternal–fetal hypothalamic–pituitary–adrenal (HPA) axis. It has been suggested that placental endocrine dysfunction or maternal stress at critical times during fetal development may influence the fetal HPA axis, leading to neuroendocrine abnormalities that could increase the child more susceptibility to neurobehavioral disorder later in life [3, 48]. Also maternal exposure to environmental stressors including noise, air pollution, heat, and traffic density can aggravate the disruption of the placental clock and fetal hypothalamic–pituitary–adrenal axis [49].

However, some researchers have raised doubts about the connection between perinatal and neonatal issues and autism, questioning whether it is a direct cause. They suggest that the correlation may be influenced by birth order, as complications during these periods are more common in first-born, fourth-born, and later offspring. It has been noted that individuals in these birth orders have a higher likelihood of developing autism [50].

This study has some limitations. First, a high heterogeneity among the results of our study was the limitation of this meta-analysis. However, with subgroup analysis based on gender, this heterogeneity was resolved in males. This study included 1,412,667 participants who reported that post-term birth was a risk factor for ASD. In addition, publication bias did not occur among the results and these were strengths of the present meta-analysis.

## Conclusion

Post-term birth still occurs relatively frequently (up to 5–10%) even in developed countries. Our results showed that post-term birth is an increased risk of ASD, although high heterogeneity was found among the studies reported based on adjusted and crude forms, however, after subgroup analysis by gender, this heterogeneity disappeared among males.

## Data Availability

The datasets used and/or analyzed during the current study are available from the corresponding author on reasonable request.

## Abbreviations

ASD: Autism spectrum disorder
PRISMA: Systematic Reviews and Meta-Analysis
GRADE: Grades of Recommendation, Assessment, Development, and Evaluation
OR: Odds ratio
HPA: Hypothalamic–pituitary–adrenal
